# Editorial: Diabetes in Africa in the 21st century: the unique and important challenge of diabetes in Africa, volume II

**DOI:** 10.3389/fpubh.2023.1265439

**Published:** 2023-08-24

**Authors:** Elias S. Siraj, Åke Sjöholm, Anne E. Sumner

**Affiliations:** ^1^Eastern Virginia Medical School, Norfolk, VA, United States; ^2^Department of Internal Medicine, Gävle Hospital, University of Gävle, Gävle, Sweden; ^3^National Institute of Diabetes and Digestive and Kidney Diseases (NIDDK), NIH, Bethesda, MD, United States; ^4^National Institute of Minority Health and Health Disparities (NIMHD), NIH, Bethesda, MD, United States; ^5^Hypertension in Africa Research Team, North-West University, Potchefstroom, North-West, South Africa

**Keywords:** diabetes in Africa, diabetes phenotypes, diabetes and malaria, diabetes risk scores, rural diabetes, diabetes in underserved

Decades ago, diabetes in Africa meant mostly type 1 diabetes (T1DM), as most of the continent was leading rural and subsistent farming or pastoral life. Obesity and the associated type 2 diabetes (T2DM) were not that common and in some communities literally unheard of.

The past few decades have seen increasing urbanization & globalization, leading to rapid and dramatic changes in dietary habits and the degree of physical activity. Those changes conspired to the rapid rise in the prevalence of obesity and T2DM.

That said, diabetes in Africa has not been well-studied and well-understood. While over time our understanding of diabetes in Africa and its unique features has improved, there remains a huge gap requiring further, high quality research. There seem to be unusual types of phenotypes in both the T1DM and T2DM populations which need further elucidation and ways of identifying them, thereby enabling proper means of prevention and treatment. The spread of T2DM to the rural populations of Africa will need further study and scrutiny. Additionally, the interaction between common conditions in Africa such as malaria and diabetes are not well-understood.

It is with those thoughts in mind that we at Frontiers opened the Research Topic “Diabetes in Africa in the 21^st^ Century: The Unique and Important Challenge of Diabetes in Africa, Volume II”, to serve as the follow-up to the first volume published a couple of years ago. The papers published under this Research Topic tried to address some of those questions in their own ways.

Christensen et al. looked at the important issue of why mothers who acquire malaria during pregnancy tend to produce low birth weight offsprings who later on as adults are at increased risk for hyperglycemia. The fact that low birth weight is associated with higher risk of T2DM as adults has been confirmed by many studies (1). In a previous study, the same group (2), demonstrated that the 30 min post OGTT blood glucose values were slightly higher in offspring of mothers who had peripheral and placental malaria, though the differences in insulin resistance and insulin secretion were not significant. Given that muscles play an important role in insulin resistance, the current study looked at differences in muscle subtypes, enzymatic activity and aerobic capacity among adults whose mothers had various degrees of malaria exposure, and found no significant difference. They therefore hypothesize, rather than insulin resistance, it is compromised insulin secretion which is the major pathogenic factor. We feel that while this may be a logical conclusion, it will need bigger and focused studies to definitively confirm the high risk of hyperglycemia in adults who had malaria exposure *in utero* and what the underlying mechanisms are. Given that malaria continues to be a major morbidity in Africa, understanding how it might impact pregnancy and the long-term health of the offsprings is an important issue which need to be pursued further.

Katte et al. did a review of the enigmatic issue of T1DM phenotypes in sub-Saharan Africa. In 1985, in recognition of unique presentations of T1DM, the WHO Study Group on Diabetes Mellitus (3) introduced the name Malnutrition Related Diabetes Mellitus (MRDM) as a separate entity. Many in Africa (4, 5) were reporting at that time an insulin-requiring diabetes manifesting itself in severely malnourished children and adults. Those patients, though they seem to share many of the phenotypes of T1DM, do not develop DKA and they seem to maintain residual insulin secretion. After several years of observation, the WHO removed the independent category of MRDM, thinking it is likely a variant of T1DM and should not be seen as a separate entity. However, looking at more recent literature (6, 7), it appears some are still wrestling with the observation that many of the presumed T1DM patients in Africa continue to show unique features and phenotypes compared to those in the Western society. It is against this background that we should look at the current review article as an attempt to summarize this complex topic and open the way for future studies to better understand T1DM in Africa.

Wentzel et al. approached the variable phenotypes of diabetes in the African diaspora living in the USA, and attempted to investigate whether the traditional risk scores for T2DM are good at identifying patients whose primary pathophysiology is beta cell failure rather than insulin resistance. Researchers in Africa (7–9) have noticed for decades that a significant fraction of those classified as T2DM do not seem to have the traditional epidemiologic and clinical characteristics associated with T2DM, but rather significant beta cell dysfunction. The authors were able to separate subjects with T2DM into those with beta cell failure vs. insulin resistance using state of the art laboratory methods. They then assessed the utility of several non-invasive T2DM risk scores in detecting diabetes due to beta cell failure. The results clearly show that those scores, while reasonable at detecting those with insulin resistance, are unable to detect those with beta cell failure. Given that a sizable proportion of T2DM patients in Africa may be of the predominantly beta cell failure type, the need to develop a valid risk score appropriate to them is evident and should be an area of focus in diabetes research in Africa. Such a score will facilitate the early identification of those at risk and thus enable the prevention to full blown T2DM.

Assefa and Shifera tackled the issue of diabetes prevalence in a very underserved and marginalized community in Ethiopia. T2DM, which was once thought as a rare condition in sub-Saharan Africa, has become an important cause of morbidity and mortality (10). Most of the studies done in Africa tend to focus on urban population where the prevalence is generally much higher than the rural population. The current study focused on a highly marginalized, predominantly rural population and therefore the finding of 15% prevalence of T2DM (mostly previously undiagnosed) confirms that the diabetes epidemic in Africa is not restricted to the cities anymore. This is of huge public health significance as African countries struggle to come out of the communicable disease focus they have had for decades and embrace the need to focus on non-communicable diseases, such as diabetes, and allocate the necessary resources.

All in all, kudos to the contributors as they have done a good job of addressing many unanswered questions, though each study had its own limitations. These types of studies pave the way for further research in our ongoing quest to better understand diabetes in Africa.

## Author contributions

ES: Conceptualization, Writing—original draft, Writing—review and editing. ÅS: Writing—review and editing. AES: Writing—review and editing.
